# E-Cigarette or Vaping Product Use-Associated Lung Injury: A Severe Case That Responded to Corticosteroid Treatment

**DOI:** 10.7759/cureus.11544

**Published:** 2020-11-18

**Authors:** Ahmad Al-abdouh, Emily Phillips, Michael G Allison

**Affiliations:** 1 Internal Medicine, Saint Agnes Hospital, Baltimore, USA; 2 Psychiatry, School of Medicine, American University of the Caribbean, Cupecoy, SXM; 3 Critical Care, Saint Agnes Hospital, Baltimore, USA

**Keywords:** vaping, lung injury, corticosteroids

## Abstract

Vaping is associated with an increased risk of lung injury; however, each case of vaping-associated lung injury leads to varying degrees of lung injury, and the response to therapy can be heterogeneous. Corticosteroid use has been suggested as a treatment for lung injuries associated with vaping.

We report a case of e-cigarette or vaping product use-associated lung injury (EVALI) that resulted in acute hypoxic respiratory failure. A 20-year-old woman presented with complaints of sore throat, dry cough, shortness of breath, and pleuritic chest pain. The patient admitted to vaping regularly for the past three years. The patient was found to be severely hypoxemic with respiratory distress and was intubated shortly after her arrival at the emergency department. She was treated with a short course of corticosteroids with tapering of the dose based on her response with significant improvement, and she was extubated on the seventh day of her admission.

EVALI is a syndrome associated with severe lung injury that results in acute respiratory failure that is clinically indistinguishable from acute respiratory distress syndrome, and it is largely a diagnosis of exclusion. The use of systemic corticosteroids in treating these patients should be considered after excluding an infectious etiology.

## Introduction

The term vaping refers to the use of battery-powered electronic cigarettes (e-cigarettes) to deliver an aerosolized product to the user, and it has been increasingly associated with lung injury [1]. This alternative to traditional smoking exposes the lungs to many harmful substances such as heavy metals and volatile organic compounds [2]. Vaping of non-nicotine liquids, such as tetrahydrocannibidiol (THC), cannabidiol (CBD), and other unknown substances has been on the rise recently [3]. E-cigarette or vaping product use-associated lung injury (EVALI) is characterized by the presence of pulmonary infiltrates on imaging, the use of electronic nicotine delivery systems within the previous 90 days, and the absence of other possible causes such as infectious, cardiac, neoplastic, or rheumatologic causes, which makes it difficult to diagnose the condition on presentation [4]. The clinical presentation of a patient with EVALI includes respiratory symptoms such as shortness of breath, cough, pleuritic chest pain, hemoptysis, and may also include constitutional symptoms like fever and gastrointestinal symptoms like vomiting and diarrhea [5]. Recently, systemic corticosteroids have been used to treat EVALI patients with encouraging results [6]. We discuss the case of a young woman who presented with severe acute hypoxic respiratory failure requiring intubation due to EVALI; her condition improved rapidly after systemic corticosteroids were administered.

## Case presentation

**Figure 1:**
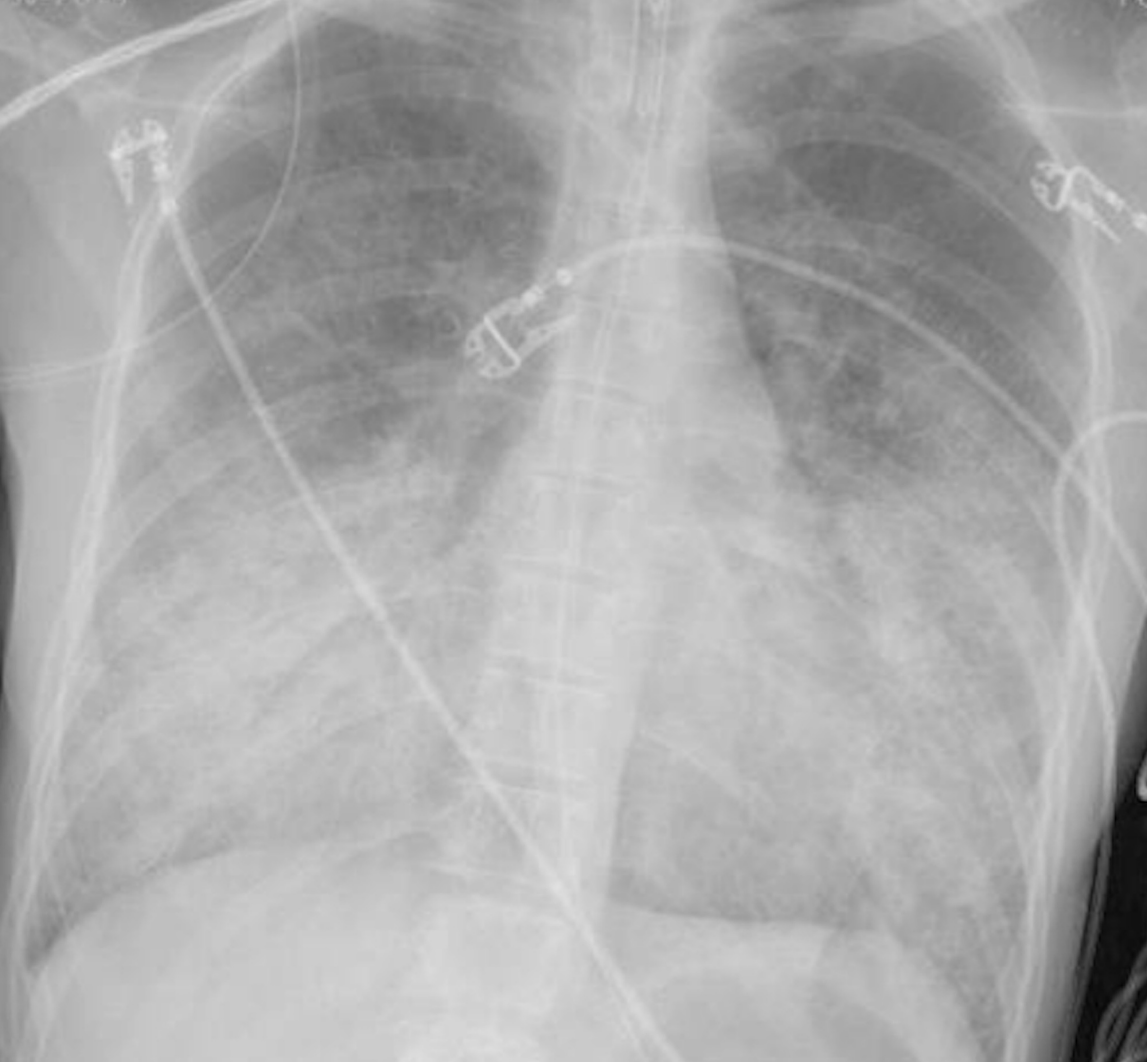
Chest X-ray on admission The image shows confluent areas of consolidation in the bilateral mid to lower lung zones

The patient was a 20-year-old female with no significant past medical history. She was transported to the emergency department via EMS from an urgent care facility due to hypoxia. The patient was complaining of subjective fever, sore throat, dry cough, nasal congestion, shortness of breath, pleuritic chest pain, non-bloody vomiting, diarrhea, and loss of appetite of five days' duration. She admitted to using e-cigarettes to vape THC-containing products and tobacco regularly with the last use being five days prior to the admission. Her temperature was 99.5 °F, heart rate was 130 beats per minute, respiratory rate was 40 breaths per minute, blood pressure was 127/73 mmHg, and she had an oxygen saturation of 88% on 15 liters per minute of oxygen via non-rebreather mask. She appeared in acute respiratory distress, and on examination, she had bilateral diffuse crepitations with good bilateral air entry. The complete blood count was remarkable for neutrophilic leukocytosis of 18.5 K/uL (normal range: 4,500-11,000 K/uL), while the serum chemistries were unremarkable. The C-reactive protein (CRP) was 83.1 mg/L (normal range: <10 mg/L), and the erythrocyte sedimentation rate (ESR) was 88 mm/h (normal range: 0-20 mm/h). The urine toxicology screen was positive for cannabinoids. The respiratory viral panel polymerase chain reaction (PCR) from a nasopharyngeal swab did not detect the presence of any virus. Extensive serological workup including *Histoplasma*, *Aspergillus*, and *Coccidioides* antibodies was negative. The urine *Legionella* and *Streptococcus pneumoniae* antigens were also negative. A chest X-ray (Figure 1) performed upon admission showed confluent areas of bibasilar consolidation.

**Figure 2:**
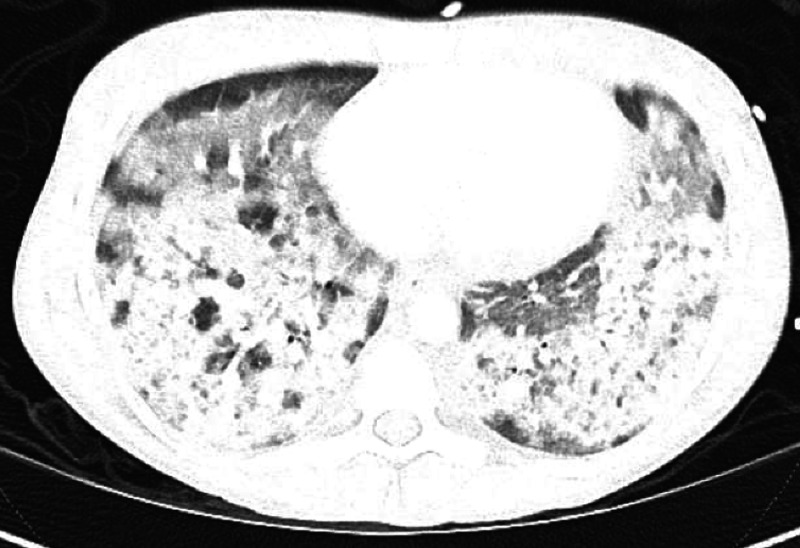
Chest CT on admission The image shows dense airspace opacities throughout the lung bases and patchy ground-glass attenuation involving each of the pulmonary lobes CT: computed tomography

A CT of the chest (Figure 2) showed dense airspace opacities throughout the lung bases and patchy ground-glass attenuation involving all pulmonary lobes.

The patient was initially put on a high-flow nasal cannula before requiring intubation and mechanical ventilation due to persistent hypoxia. The differential diagnoses in these cases include community-acquired pneumonia, acute eosinophilic pneumonia, acute interstitial pneumonia, and hypersensitivity pneumonitis. Bronchoscopy with bronchoalveolar lavage (BAL) was performed and was unremarkable. An Oil Red O stain was performed and did not show the presence of lipid-laden macrophages. The BAL cultures, including bacterial, fungal, mycobacterial, viral, *Mycoplasma*, and *Legionella* cultures did not show any growth. She was treated with a five-day course of broad-spectrum antibiotics including vancomycin, azithromycin, piperacillin-tazobactam, and oseltamivir. On the second day of admission, she was started on systemic corticosteroids (methylprednisolone 1 mg/kg/day) owing to the worsening of her condition, and due to the fact that the previously mentioned cultures had not shown any growth after 24 hours. Her response to treatment was suboptimal with a continuing need for mechanical ventilation and persistent hypoxemia. The dose of systemic corticosteroids was increased on the fourth day (methylprednisolone 2 mg/kg/day), and she subsequently started to improve and was extubated on the seventh day of admission. Table 1 chronicles the timeline of events regarding the patient's hospital course.

**Table 1:** Timeline of events BAL: bronchoalveolar lavage

Timeline	Events
Day 1	[] Initial presentation [] Starting of broad-spectrum antibiotics and oseltamivir [] Intubation and mechanical ventilation [] Bronchoscopy with BAL: no endobronchial lesions or significant secretions
Day 2	[] Methylprednisolone was started (1 mg/kg/day)
Day 4	[] Cisatracurium initiated due to hypoxia, tachypnea, and high oxygen requirements with a P/F of <150 [] Methylprednisone dose was increased (2 mg/kg/day)
Day 5	[] Antibiotics and oseltamivir were stopped
Day 8	[] Extubated to high-flow nasal cannula
Day 9	[] Downgraded from ICU to the floor [] Switched to prednisone (1 mg/kg/day) orally
Day 11	[] Prednisone was stopped
Day 13	[] Discharged home

This case was unique as it was a severe form of EVALI even though it was not associated with lipid-laden macrophages on oil red staining of BAL specimen, and showed a dramatic improvement after increasing the dose of systemic corticosteroid treatment.

## Discussion

We discussed a case of severe EVALI that responded well to a short course of systemic corticosteroids. The patient was diagnosed to have EVALI as she had a history of vaping, in the absence of any other cause to explain her acute hypoxic respiratory failure and lung infiltrates. Since it is a diagnosis of exclusion, it is important to perform a full workup including serological tests, cultures, and imaging and may also require bronchoscopy. 

The definitive cause of lung injury in EVALI is unknown, but it is noteworthy to mention that vitamin E acetate, which is commonly used in e-cigarettes or vaping products, has been detected in the BAL fluids of most patients diagnosed with EVALI [7]. This provides evidence that these devices can deliver vitamin E acetate to epithelial-lining fluids but is not proof that vitamin E acetate is the cause of the lung injury. It has also been reported that EVALI patients usually have a history of using THC-containing products at higher rates, and this has prompted the Centers for Disease Control and Prevention (CDC) to recommend not using e-cigarettes or vaping products that contain THC [8].

EVALI has a variety of imaging patterns, which suggests different mechanisms of injury, but most patterns have basilar-predominant consolidation, ground-glass opacity, and areas of lobular or subpleural sparring [9]. Our patient's chest X-ray and CT scan were consistent with the basilar pattern, which supported our diagnosis. Another feature that has commonly been noted in previous reports [10], but was absent in this case, is the presence of lipid-laden macrophages seen with Oil Red O, which is suggestive of lipoid pneumonia.

Treatment of EVALI usually includes empiric antibiotics to cover for community-acquired pneumonia, antivirals especially during the influenza season, systemic corticosteroids, supportive care, and vaping cessation [11]. Since most of the deaths occur due to acute respiratory distress syndrome, it is important to provide optimal supportive care as mechanical ventilation of these patients can be complicated. They are at risk of developing barotrauma from mechanical ventilation and hence using a lower tidal volume with varying levels of positive end-expiratory pressure is recommended. Patients may also need neuromuscular blockade, prone positioning, and extracorporeal membrane oxygenation if not sufficiently ventilated [12].

The role and efficacy of corticosteroids in the treatment of lung injury induced by vaping could be attributed to inflammatory response suppression [11]. There is no consensus on a specific dose, duration, or taper plan, and these factors usually depend on the patient’s response to the treatment. We initially withheld corticosteroids while evaluating the patient for infectious etiologies, lest it might get aggravated by corticosteroid treatment. We started corticosteroids with a lower dose when her clinical condition necessitated the use of mechanical ventilation, and since her cultures had not shown any growth. Subsequently, the corticosteroid dose was increased due to a suboptimal response. We believe that increasing the systemic corticosteroid dose in this case definitely changed the patient’s course of EVALI as she improved shortly after this dosage increase. This is consistent with the current evidence of using corticosteroids in EVALI [13].

This case is worth studying because it highlights the typical findings that clinicians need to look out for in diagnosing these patients. Our patient had neutrophilic leukocytosis, an increase in inflammatory markers, no growth of microorganisms on cultures, and classical imaging findings. The absence of common oil red staining of macrophages on the BAL specimen does not rule out EVALI, and this is another distinguishing feature of this case. The patient required intubation and mechanical ventilation for more than a week and responded well after increasing the corticosteroid dose. This impressive response to a higher dose of corticosteroids (methylprednisolone 2 mg/kg/day) may help in shaping the future dosage recommendations related to systemic corticosteroid use in EVALI patients.

## Conclusions

The use of e-cigarettes and vaping is associated with lung injury and is usually a diagnosis of exclusion. These patients can present with a variety of signs and symptoms, but will mostly have respiratory symptoms. Systemic corticosteroid use in treating these patients should be considered after excluding an infectious etiology with subsequent tapering of the dose based on clinical response.
